# Recent advances in PTP1B signaling in metabolism and cancer

**DOI:** 10.1042/BSR20211994

**Published:** 2021-11-12

**Authors:** Olga Villamar-Cruz, Marco A. Loza-Mejía, Luis E. Arias-Romero, Ignacio Camacho-Arroyo

**Affiliations:** 1 Unidad de Investigación en Reproducción Humana, Instituto Nacional de Perinatología-Facultad de Química, Universidad Nacional Autónoma de México (UNAM), Ciudad de México, Mexico; 2 Unidad de Investigación en Biomedicina (UBIMED), Facultad de Estudios Superiores-Iztacala, UNAM. Tlalnepantla, Estado de México 54090, Mexico; 3 Facultad de Ciencias Químicas, Universidad La Salle-México. Benjamin Franklin 45 06140, Mexico City, Mexico

**Keywords:** Cancer, Protein Tyrosine Phosphatase 1B, Receptor Tyrosine Kinase, Signaling, Small molecule inhibitor

## Abstract

Protein tyrosine phosphorylation is one of the major post-translational modifications in eukaryotic cells and represents a critical regulatory mechanism of a wide variety of signaling pathways. Aberrant protein tyrosine phosphorylation has been linked to various diseases, including metabolic disorders and cancer. Few years ago, protein tyrosine phosphatases (PTPs) were considered as tumor suppressors, able to block the signals emanating from receptor tyrosine kinases. However, recent evidence demonstrates that misregulation of PTPs activity plays a critical role in cancer development and progression. Here, we will focus on PTP1B, an enzyme that has been linked to the development of type 2 diabetes and obesity through the regulation of insulin and leptin signaling, and with a promoting role in the development of different types of cancer through the activation of several pro-survival signaling pathways. In this review, we discuss the molecular aspects that support the crucial role of PTP1B in different cellular processes underlying diabetes, obesity and cancer progression, and its visualization as a promising therapeutic target.

## Introduction

Protein tyrosine phosphorylation is one of the most important post-translational modifications in eukaryotic cells and plays a fundamental role in cellular physiology, since it can promote allosteric changes essential for protein–protein interactions, protein stability, the proper protein localization, and the enzymatic activity [1,2]. Reversible tyrosine phosphorylation of proteins is reciprocally controlled by protein tyrosine kinases (PTKs) and protein tyrosine phosphatases (PTPs). Consequently, the aberrant activity of these enzymes has been linked to various diseases including cancer [3,4]. PTPs are hydrolases that dephosphorylate phosphotyrosine (pTyr) residues of a large number of proteins producing a dephosphorylated protein at tyrosine residues and inorganic phosphate [5].

In the human genome, the PTP superfamily is divided into four separate families (Class I, II, III, and IV) according to differences in the amino acid sequence at their catalytic domains and the nucleophilic amino acid used in the catalytic reaction. Classes I, II, and III are formed by cysteine (Cys)-based PTPs and Class IV by aspartate (Asp)-based PTPs (Table 1) [5,6]. The largest family is the Class I Cys-based PTPs, which can be further divided into two subfamilies: tyrosine-specific or classical PTPs, which includes receptor-type PTPs (PTPRs) and non-receptor PTPs (NRPTPs); and vaccinia virus H1-like (VH1-like) dual-specificity phosphatases (DUSPs), which are the more diverse group. In terms of substrate specificity, besides pTyr, they have the ability to dephosphorylate phosphoserines (pSer), phosphothreonines (pThr), and/or phosphoinositides (PIPs), in addition to pTyr [7,8].

**Table 1:** Classification of human PTPs

			M.W. (kDa)	Substrate
**Class I**	(Cys)-based	Classical PTPs	48–213	pTyr, PIPs
		VH1-like	21–50	pTyr, pSer, pThr, PIPs, Phosphatidylglycerophosphate, Phosphoglycogen, 5′ CAP-mRNA
**Class II**	(Cys)-based	LMPTP	18	pTyr
**Class III**	(Cys)-based	CDC25	59–65	pTyr, pThr
**Class IV**	(Asp)-based	EYA	59–70	pTyr, pSer

The Class II Cys-based PTP is represented in the human genome by a single gene termed *ACP1*, which encodes the 18-kDa low molecular weight phosphatase LMPTP, a protein involved in the modulation of various signaling pathways including T-cell receptor activation [9,10]. The Class III Cys-based PTPs comprises three cell cycle regulators, CDC25A, CDC25B and CDC25C, which regulate cell cycle progression and checkpoint pathways that control the DNA damage response, mainly by activating the cyclin-dependent kinases (Cdks) through dephosphorylation of the Thr^14^ and Tyr^15^ residues in their ATP-binding loop [11,12]. Finally, the Class IV Asp-based PTPs in humans is represented by the four members of the EYA family (EYA1–4), a group of proteins originally identified as essential transcriptional co-activators of the Six family of homeoproteins [13]. The highly conserved C-terminal domains of the EYA proteins were discovered to act as an Mg^2+^-dependent Tyr phosphatases, making EYAs the first transcriptional activators to harbor intrinsic phosphatase activity [14]. So far, the only validated substrate identified for the Tyr phosphatase activity of EYAs is the histone H2AX [15]. In the next sections, we will focus on PTP1B, a Class I phosphatase that plays a promoting role in the development of different types of cancer through the activation of pro-survival signaling pathways.

## PTP1B structure and regulation

**Figure 1:**
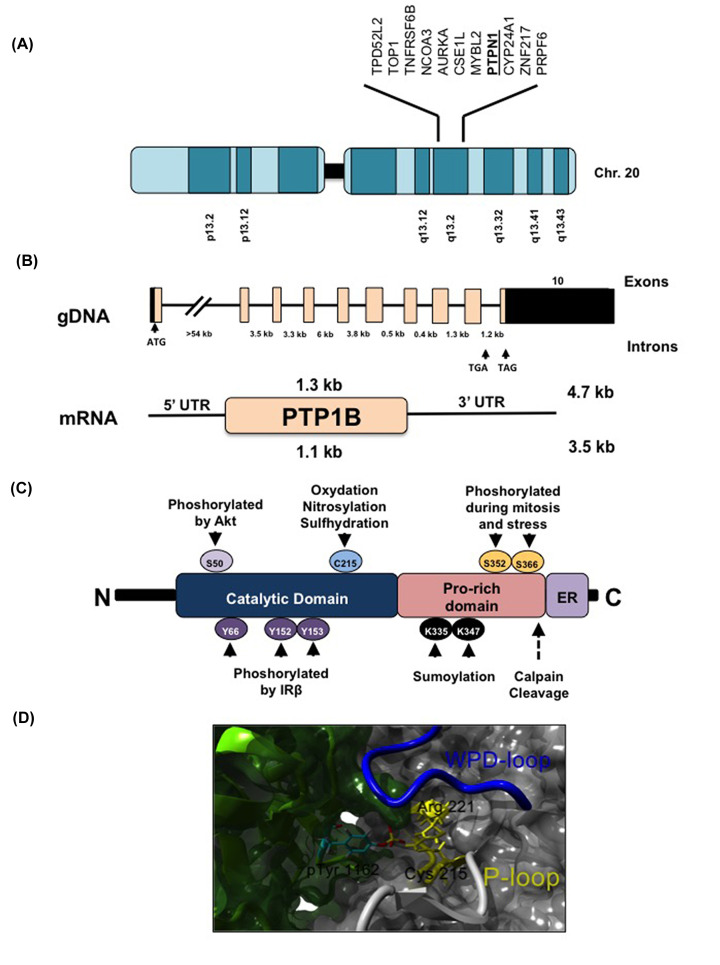
Genomic organization and structure of PTP1B (**A**) Graphical representation of the human chromosome 20. The human PTP1B gene is located into the chromosome 20q13.1, a commonly amplified region in breast and ovarian cancers. (**B**) The upper panel shows the schematic representation of the human PTP1B gene. Exons are boxed, and the black region indicates the coding sequence. The two alternative termination codons, one in intron 9 and the other in exon 10, are indicated. The lower panel shows the schematic representation of PTP1B mRNA. In humans there are two transcripts, a larger 4.7 kb one, which includes intron 9, and a smaller 3.5 kb transcript with the intron 9 spliced out. (**C**) Structural domains and post-translational modifications of PTP1B. (**D**) Model of the PTP1B (gray)—Insulin Receptor (green) interaction predicted by protein–protein docking using ClusPro. Interaction of the phosphotyrosine 1162 of IR with the catalytic residues Cys^215^ and Arg^221^ of PTP1B is highlighted. Abbreviation: IR, insulin receptor.

PTP1B is a ubiquitous and abundant intracellular enzyme and the prototype of non-receptor phosphatases. It was the first enzyme of its class to be purified to homogeneity [16,17], cloned [18,19], and crystallized [20,21]. Most discoveries about the mechanisms of regulation, structure, and function of this class of phosphatases were described in PTP1B [22]. This enzyme is encoded by the protein tyrosine phosphatase non-receptor type 1 (*PTPN1*) gene. In humans, it is located at chromosome 20q13.1 (Figure 1A), in a region that has been associated with diabetes and obesity, and spans over 74 kb with an unusually large first intron. In normal human cells, two PTP1B mRNAs were identified, a larger 4.7 kb transcript which includes intron 9, and a smaller 3.5 kb transcript with intron 9 spliced out (Figure 1B) [23]. Interestingly, this genetic locus is commonly amplified in breast cancer and has been associated with poor prognosis [24].

The *PTPN1* gene is translated into a 435-amino acid protein composed of an N-terminal catalytic domain (residues 30–278), two proline-rich domains (residues 278–401), at least one of which is crucial for protein–protein interactions, and a 35 amino acid C-terminal tail (residues 401–435) that targets PTP1B to the cytoplasmic face of the endoplasmic reticulum (ER) (Figure 1C) [25,26]. Despite its ER localization, PTP1B can interact with diverse substrates, which include cytosolic, nuclear, plasma membrane-bound, mitochondrial, and adherent junction proteins [27–29]. The non-catalytic motifs and the catalytic domain regulate the interaction between PTP1B and its substrates. PTP1B contains proline-rich motifs that mediate its association with several Src-homology 3 (SH3) domain-containing substrates such as p130^Cas^ and Src [30,31]. In addition, PTP1B catalytic domain has a high specificity and preference towards certain substrates. Crystallographic analysis of PTP1B interaction with the insulin receptor (IR) established that the sequence E/D-pY-pY-R/K is needed for optimal substrate recognition. Indeed, PTP1B exhibits higher affinity (∼70 fold) for tandem pTyr containing peptides compared with those with mono-pTyr (Figure 1D) [32]. Remarkably, this motif has been used to predict novel PTP1B substrates such as Janus Kinase 2 (JAK2) and Tyrosine Kinase 2 (TYK2) [33]. Although the tandem pTyr-motif is important for optimal substrate recognition it is not a requirement, as several well-established PTP1B substrates do not possess this sequence.

The regulation of PTP1B expression at transcriptional and post-transcriptional levels or by post-translational modifications has been extensively studied since its discovery. The promoter regions of human and mouse PTP1B genes lack a TATA-box, but are rich in GC sequences, and possess binding sites for a wide variety of transcription factors such as Sp1, Early Growth Response Protein 1 (EGR1), Nuclear Factor κ B (NF-κB), Y box binding protein 1 (YBX1), and the androgen receptor (AR) among others [34–37]. Recently, it has been reported that PTP1B expression can also be regulated at post-transcriptional level through the interaction of multiple miRNAs such as miR-338-3p, miR-193a-3p, miR-135a, miR-146-b, and miR-206 with its 3′-UTR region [38–43].

At post-translational level, PTP1B function is regulated by several modifications including oxidation, nitrosylation, sumoylation, phosphorylation, and proteolytic cleavage (Figure 1C) [44]. Like other classical PTPs subfamily members, PTP1B is susceptible to oxidation by reactive oxygen species (ROS) [45]. Oxidation of PTP1B Cys^215^ suppresses its activity by converting the sulphenic acid (S–OH) into a cyclic sulphenamide, in which the sulphur atom of the catalytic Cys^215^ is covalently linked to the main chain nitrogen of an adjacent residue. This oxidation is accompanied by significant conformational changes in the architecture of the active site that inhibit substrate binding [46,47]. These structural changes are reversible and protect the enzyme from higher order (S–O_2_H and S–O_3_H) irreversible oxidation, and facilitate the reduction back to its active form [47]. Similar to ROS, reactive nitrogen species (RNS) suppresses PTP1B activity [48]. In particular, *S*-nitrosylation prevents PTP1B Cys^215^ from subsequent oxidation when subjected to oxidative stress, suggesting that this post-translational modification protects PTP1B from permanent inactivation caused by oxidative stress [49]. PTP1B is sumoylated in the residues Lys^335^ and ^347^, and its C-terminal tail is required for maximum sumoylation [50]. In addition, sumoylation also affects PTP1B-mediated dephosphorylation of its nuclear substrate, emerin, a protein involved in the maintenance of the nuclear architecture [29]. Ser and Tyr phosphorylation can also regulate PTP1B activity. For instance, insulin promotes PTP1B phosphorylation at Tyr^66^, ^152^, and ^153^. It is possible that the phosphorylation of Tyr^152^ and ^153^ is more important for its effects on protein interaction since the mutation of these residues markedly impairs the association of PTP1B with activated IR [51,52]. Besides, PTP1B is phosphorylated at multiple Ser residues during mitosis [53]. Moreover, phosphorylation of PTP1B at Ser^50^ by Akt inhibits PTP1B activity and impairs its ability to dephosphorylate the IR, possibly as a positive feedback mechanism to potentiate insulin signaling [54]. Finally, PTP1B activity can also be regulated by proteolytic cleavage. In activated platelets, PTP1B is released from the ER by calpain-cleavage, generating a cytoplasmic constitutively active enzyme [55]. The diversity of PTP1B post-translational modifications, illustrate the complexity of its regulation.

## Major signaling pathways regulated by PTP1B and their role in metabolism

**Figure 2:**
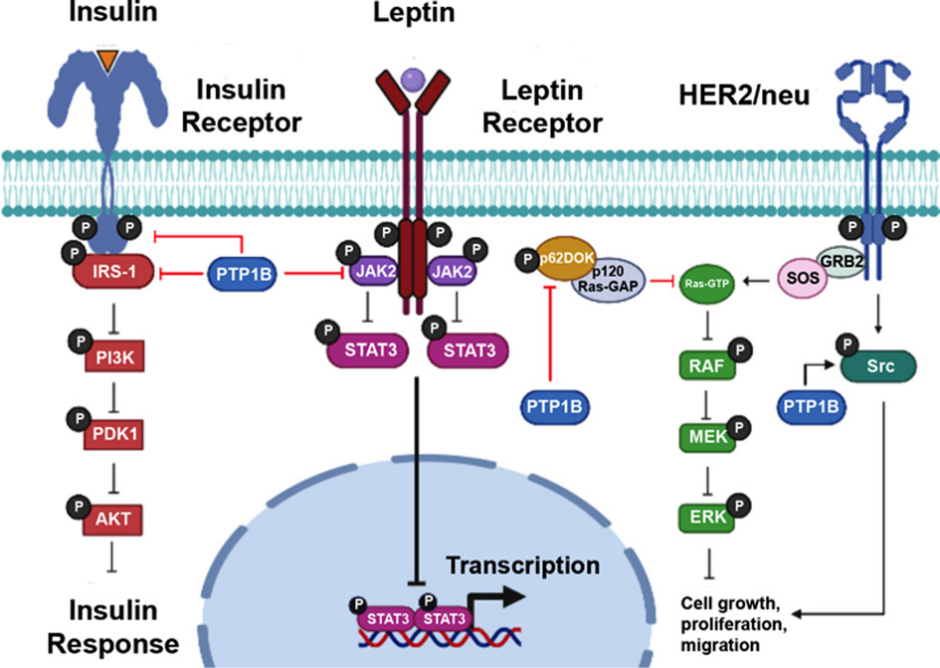
Major signaling pathways regulated by PTP1B PTP1B exerts a negative role in the insulin and leptin signaling, whereas it has a promoter role on Her2/Neu-induced tumorigenesis. IR activation promotes tyrosine phosphorylation of IRS-1, leading to PI3K and Akt activation. PTP1B attenuates insulin signaling following receptor endocytosis, and it dephosphorylates IRS-1. Activation of the LR leads to tyrosine phosphorylation of JAK2 and subsequent activation of STAT3-mediated transcription. JAK2-STAT3 activity is also negatively regulated by PTP1B. Regarding the oncogenic role of PTP1B signaling, it dephosphorylates p62Dok, which then inhibits p120RasGAP, thereby promoting Ras-MAPK-mediated cell proliferation. Similarly, PTP1B dephosphorylates Src at Y529 to promote cell migration and enables the subsequent activation of small GTPases such as Ras and Rac1. Abbreviation: LR, leptin receptor.

During the last 25 years, several reports have shown that PTP1B acts as a critical negative and positive regulator of numerous signaling cascades (Figure 2). Initial *in vitro* evidence suggested that PTP1B was a negative regulator of the IR and its downstream signaling pathways [17]. Confirmation of the importance of PTP1B in metabolism came from PTP1B knockout (KO) mice, which display lower blood glucose and insulin levels than their wildtype (WT) littermates. In addition, when the PTP1B KO and heterozygous mice were subjected to a chronic hypercaloric diet, they remained resistant to weight gain and insulin-sensitive. In contrast, the WT mice rapidly gained weight and developed insulin resistance [56]. At the molecular level, PTP1B KO mice exhibit increased phosphorylation of the IR in liver and muscle tissue upon insulin injection, establishing PTP1B as a key regulator of insulin signaling. Further studies using tissue-specific PTP1B KO animals revealed that despite improved glucose homeostasis and insulin signaling in muscle and liver, these animals gained weight and developed insulin resistance when they were subjected to a high-fat diet [57]. In contrast, neuron-specific PTP1B KO mice were protected from diet-induced obesity and glucose intolerance, indicating that neuronal PTP1B regulates body weight, adiposity, and leptin signaling [58]. Mechanistically, PTP1B dephosphorylates the IR at the Tyr residues 972, 1162, and 1163 [32,51]. In addition, it has been shown that in the liver of hyperglycemic Insulin Receptor Substrate-2 (IRS-2) deficient mice, PTP1B impairs IR/IRS-1-mediated insulin signaling. Accordingly, deletion of PTP1B promotes insulin sensitivity in the liver these mice through the restoration of IRS-1-mediated PI3K/Akt/Foxo1 phosphorylation and the inhibition of gluconeogenic enzymes [59].

Besides its prominent role in insulin signaling, PTP1B has been linked to leptin regulation. The first evidence that PTP1B attenuates leptin signaling came from PTP1B KO mice. In this animal model, PTP1B is able to dephosphorylate JAK2, a well-known downstream effector of the leptin receptor (LR), resulting in an inhibition of the synthesis of the appetite-stimulating hormone, neuropeptide Y. Moreover, mice lacking both leptin and PTP1B, exhibit reduced weight gain, low amounts of adipose tissue and high resting metabolic rates [36,60]. These effects are mediated by a PTP1B-dependent mechanism for the control of diet-induced obesity [58]. It has been reported that in the brain, there are two distinct populations of first-order neurons, which synthesize two different proteins, agouti-related protein (AgRP) or proopiomelanocortin (POMC), with opposite effects on energy balance [61,62]. Mice with a POMC neuron-specific deletion of PTP1B are thin, even in the presence of a hypercaloric diet, and exhibit improved leptin sensitivity and increased energy expenditure compared with WT mice [63]. However, there were no differences in food intake between WT and PTP1B KO animals, suggesting that PTP1B function is important in other neuronal populations regulating food intake.

Since inflammation and activation of the immune system are involved in the pathogenesis of obesity-related insulin resistance and type 2 diabetes, and PTP1B modulates insulin and leptin signaling through the regulation of JAKs and STATs, it has been suggested that there is a link between PTP1B and immune signaling. The expression of cytokines, such as the Tumor Necrosis Factor α (TNFα), Interleukin (IL)-1 (IL-1) and IL-6, is part of the body’s response to obesity-induced metabolic changes during inflammation [64]. Clear links between PTP1B and several of immune pathways have been established, including the regulation of the JAK-STAT pathway [33], as well as TNFα and IL-6 signaling [65,66]. Therefore, it is feasible to assume that PTP1B is directly involved in the regulation of obesity through its action on both IR and leptin signaling, and indirectly through of the modulation of the inflammatory response and recruitment of immune cells.

## PTP1B in cancer

The role of PTP1B in cancer has been extensively reviewed [22,44,67,68]. In the last decade several reports pointed out that PTP1B plays a key role in cancer, serving as both tumor suppressor and tumor promoter depending on the cellular context. Here, we will briefly discuss some of the most recent findings regarding PTP1B signaling in cancer.

The tumor suppressing properties of PTP1B were initially attributed to observations from cell culture experiments demonstrating that its overexpression prevents cell transformation and decreases tumorigenicity in v-Src [26] and Neu (the rat ortholog to the human epidermal growth factor receptor 2 [Her2]) [18] transformed fibroblasts. In addition, PTP1B also suppresses Bcr-Abl transformation in Rat-1 cells [69]. Despite these initial observations, two independent studies confirmed that PTP1B acts as an oncogene in a mouse model of Neu-driven breast cancer [70,71]. What is not completely understood, however, is the mechanism by which PTP1B cooperates with Neu during the transformation of breast epithelial cells. Two potential substrates, p62Dok and Src, have been proposed to mediate the oncogenic signaling effects of PTP1B, but no systematic evaluation has been carried out to confirm whether these proteins explain the function of PTP1B in Neu signal transduction. Regarding p62Dok, it has been established that in breast and colon cancer, it inhibits p120RasGAP when it is dephosphorylated by PTP1B, thus leading to elevated levels of GTP-bound Ras [72]. In immortalized mouse embryonic fibroblasts (MEFs) lacking PTP1B, there is an increase in p62Dok tyrosine phosphorylation levels, which promotes the activation of p120RasGAP and the consequent inhibition of Ras signaling [73]. Consistent with this *in vitro* evidence, Julien et al*.* observed enhanced p62Dok phosphorylation in PTP1B-deficient tumors, correlating with diminished p120RasGAP phosphorylation in a Neu transgenic mouse model [71]. Other possible PTP1B target is Src, however, changes in Src phosphorylation were not reported in either of the aforementioned tumor models. Nevertheless, recent *in vitro* evidence showed that in a 3D cell culture setting, PTP1B-mediated Src activation is a prime target in Her2 (Neu)-induced human breast cancer cells. In these studies, activated Src bypasses the requirement of PTP1B for Her2-mediated transformation. Furthermore, exogenous expression of PTP1B, but not a catalytically active PTP1B mutant that cannot bind or activate Src, recapitulated the effects of activated Her2 on the architecture of the 3D spheroids, including loss of luminal apoptosis and maintenance of cell proliferation [74].

Although the oncogenic role of PTP1B has been extensively studied in breast cancer, some evidence demonstrates that PTP1B plays an important role in the development of some other types of cancer. PTP1B is highly overexpressed and it has been associated with tumor stage and overall survival of non-small cell lung cancer (NSCLC) patients. In this model, PTP1B dephosphorylates the Tyr^527^ of Src with the subsequent activation of the Ras/RAF/MEK/Erk pathway, promoting cell proliferation and metastasis in an Src-dependent manner [75]. In a recent study, splice mutations of PTP1B were found in approximately 20% of the human colon and thyroid cancer specimens analyzed. Importantly, the splice variant PTP1BΔE6, which is expressed in colon and thyroid tumors, lacks phosphatase activity. However, its expression induced transformation of rat fibroblasts without Src activation, indicating that it is involved in signaling pathways independent of Src [76].

PTP1B is highly expressed in pancreatic ductal adenocarcinoma, and its expression is correlated with distal metastasis and tumor staging. Mechanistically, PTP1B regulates the PKM2/AMPK/mTOC1 signaling pathway to regulate cell growth. PTP1B inhibition significantly increased the phosphorylation of PKM2 at the residue Tyr^105^, resulting in the activation of AMPK which in turn decreased mTOC1 activity and led to the inhibition of p70S6K, inducing cell death [77]. Interestingly, there is evidence that PTP1B acts as a tumor suppressor in NSCLC, and in some other tumors, such as esophageal cancer, lymphoma, glioblastomas, and melanoma [78–82]. The molecular mechanism by which PTP1B acts as a tumor suppressor has only been described in detail in melanoma and glioblastoma. In melanoma, PTP1B dephosphorylates caveolin and down-regulates an Rab5/Rac1 signaling axis, which is essential in cell migration. In glioblastomas, PTP1B knockdown or treatment with a PTP1B small-molecule inhibitor, causes a significant decrease in IL-13-mediated adhesion, migration and invasion by inhibiting the dephosphorylation of Src and consequently, the inactivation of the Src/Ras/Raf/MEK/ERK and PI3K/AKT pathways [81]. The tumor suppressing function of PTP1B in NSCLC, esophageal cancer and lymphoma remains unknown. Thus, the dual role of PTP1B in cancer is tissue-specific. Finally, a recent study showed that PTP1B is overexpressed in melanoma, where it promotes migration and metastasis by binding Src and dephosphorylating the inhibitory site Tyr^530^, thus leading to the activation of this kinase [82].

## Recent advances in PTP1B inhibitor development

**Figure 3:**
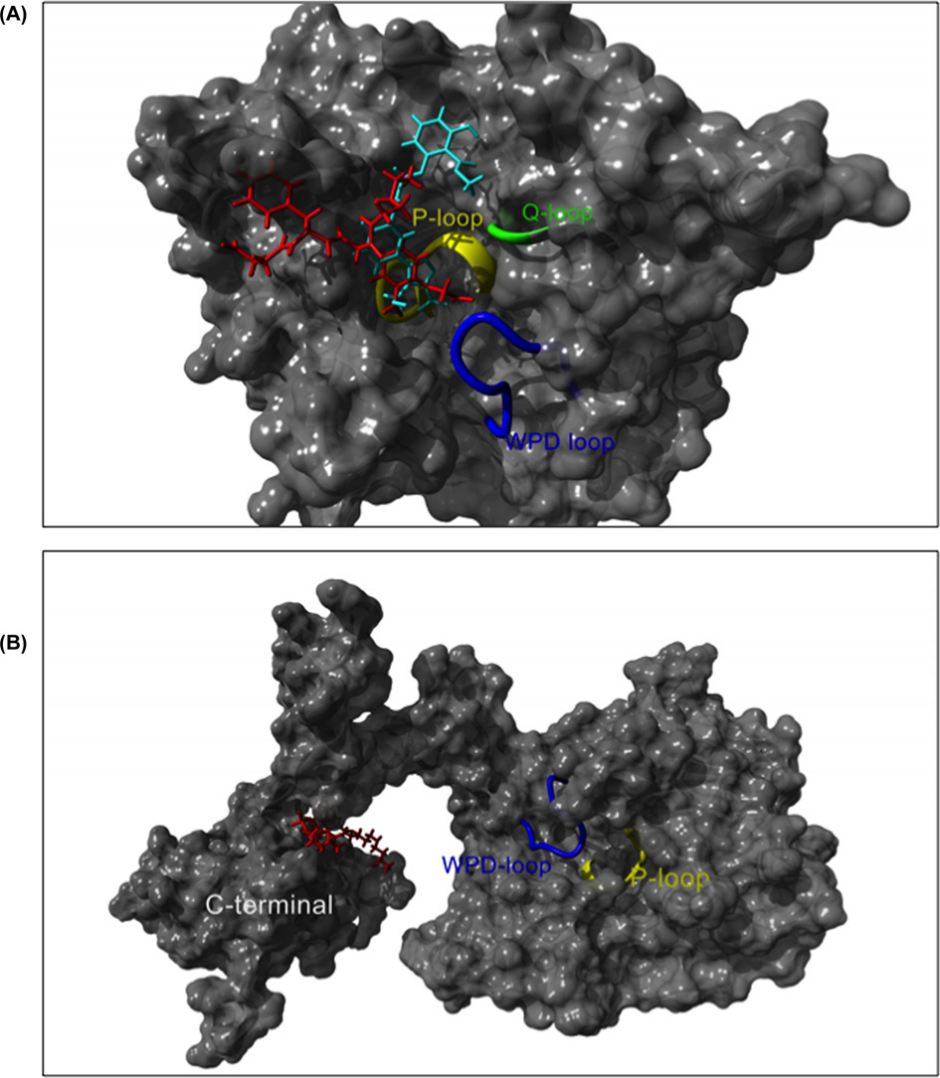
Schematic representation of PTP1B interaction with competitive and allosteric inhibitors (**A**) Small molecule phosphotyrosine mimetics like PNU 179326 (in red, PDB code: 1G7G) and DB08001 (in cyan, PDB code: 1XBO) are competitive inhibitors of PTP1B. These molecules usually bind between the WPD loop and P-loop. (**B**) Predicted interaction of the allosteric inhibitor trodusquemine (in red) in the C-terminal domain of PTP1B.

The implication of PTP1B in several pathological conditions, varying from metabolic disorders to cancer, has placed it under the spotlight as being a promising therapeutic target. However, the identification of selective PTP1B small molecule inhibitors with appropriate pharmacokinetic properties has proven extremely challenging. Many competitive PTP1B inhibitors are phosphotyrosine mimetics that contain a carboxylic or phosphonic acid and a large lipophilic tail in order to maximize the binding energy of the enzyme–inhibitor complex (Figure 3A) [83]. These small molecule inhibitors generally possess low ligand efficiency, poor cell permeability, low bioavailability, and suboptimal pharmacokinetic properties. To overcome these challenges, several approaches have been used, including the application of acidic isosteres, which have improved some of the absorption, distribution, metabolism and elimination (ADME) properties, and the design of bidentate ligands, which bind not only to the catalytic site, but also to an allosteric site or side pockets as well. This strategy allowed the development of more potent inhibitors with enhanced selectivity towards PTP1B over other PTPs [84]. In addition, trodusquemine (MSI-1436), a cholesterol metabolite originally identified in the liver of the dogfish shark *Squalus acanthia*, directly targets the C-terminus region of PTP1B blocking its phosphatase activity (Figure 3B) [85]. As trodusquemine showed promising effects in cellular and animal models, other derivatives have been developed including claramine, a polyaminosteroid which is easier to synthesize and showed similar effects than trodusquemine, restoring glycemic control in diabetic mice as determined by glucose and insulin tolerance tests [86]. More recently, *in vivo* studies using high fat diet-induced obese mice treated with the chemical compound DPM-1001, a potent, specific, and orally bioavailable analog of trodusquemine, showed a significant reduction in fat and insulin levels as a consequence of improved energy metabolism in the obese animals [87]. Despite these efforts, none of the available inhibitors have made their way through to Phase II clinical trials yet. However, in the last decade, some of these molecules have been used to generate more potent PTP1B inhibitors. In addition, some other chemical compounds such as Fumosorinone (FU), and Mulberrofuran decreased PTP1B expression attenuating its effects (Table 2) [88–89]. Moreover, several promising PTP1B inhibitors have been developed, although their efficacy has only been tested *in vitro* (Table 2) [90–95].

**Table 2:** Examples of PTP1B inhibitors

Drug	Effect	Reference
FU	FU increases glucose uptake in insulin-resistant HepG2 cells and decreases the expression of PTP1B	[88]
Mulberrofuran	Enhances glucose uptake significantly and decreases PTP1B expression in a dose-dependent manner	[89]
Infliximab	Restores phosphorylation of IRS-2 and Akt by attenuating PTP1B activation	[90]
Tanshinones derivatives	Potent PTP1B inhibitory activity *in vitro*	[91]
Benzoylsulfonamide derivatives	Selective inhibitory activity against PTP1B *in vitro*	[92]
Oleanolic acid derivatives	Enhanced inhibitory effect over PTP1B activity by increasing molecular interactions with either catalytic or allosteric sites	[93]
Trivaric acid	Potent PTP1B inhibitory activity *in vitro*	[94]
Asperentin B	Selective inhibitor of PTP1B *in vitro*	[95]
1,2-Naphtho-quinine derivative	Selective inhibitory activity against PTP1B *in vitro*	[96]

Another strategy to modulate PTP1B activity has been its genetic targeting using antisense oligonucleotides (ASOs) that bind PTP1B mRNA reducing protein translation. Swarbrick et al. investigated the effect of inhibiting of PTP1B with the subcutaneous administration of the PTP1B ASO, ISIS 113715, in monkeys. ISIS 113715 improved insulin sensitivity during a glucose tolerance test, and caused a rise in adiponectin levels [96]. Furthermore, PTP1B ASO ISIS113715 was advanced into Phase II clinical trials and shown to reduce plasma glucose and LDL-cholesterol in diabetic patients without causing weight gain [97]. Development of ASO ISIS113715 has been reportedly suspended and replaced by the new ASO PTP1BRX. Currently the Phase II trial for diabetes is complete (IONIS PTP1BRX, Ionis Pharmaceuticals, https://clinicaltrials.gov/ ID NCT01918865). The results of this clinical trial showed that in combination with metformin alone or with a sulfonylurea, PTP1BRX improved glycemic parameters and suppressed leptin signaling [98].

## Conclusions

It is clear that PTP1B signaling plays an important role in the pathogenesis and development of a wide array of human diseases. Efforts to block PTP1B activity directly are ongoing, based on structural considerations and on a better understanding of PTP1B processing and cellular localization. In parallel, new ways of identifying critical effectors of PTP1B for malignant transformation are being evaluated. While Src and Ras/Raf/MEK/ERK signaling represent the best characterized pathways used during tumor development, several additional PTP1B effectors such as the IR, IRS-1, and JAKs/STATs have been implicated in type 2 diabetes, obesity, and interestingly in the modulation of immune response, thus representing a very attractive and complicated therapeutic target.

There is accumulating evidence indicating that the function and signaling pathways regulated by PTP1B are tissue- and cell-specific, and that the general principles of PTP1B function defined by *in vitro* methods or from one cell type may not apply to another cell type *in vivo*. Future mouse genetic studies of PTP1B, combined with specific regulator and effector knockout studies, will be useful to better understand specific signaling pathways regulated by this phosphatase.

Finally, we are now at the beginning of understanding the diverse roles of PTP1B and the promising effects of PTP1B inhibitors in human diseases. In the future we will have a better understanding of the multifunctions and the complex regulatory mechanism of PTP1B, which could be of great significance in the development of more specific targeted therapeutic strategies.

## Abbreviations

ASO: antisense oligonucleotide
ER: endoplasmic reticulum
Her2: human epidermal growth factor receptor 2
IL: interleukin
IR: insulin receptor
IRS: insulin receptor substrate
JAK: Janus Kinase
KO: knockout
NSCLC: non-small cell lung cancer
POMC: proopiomelanocortin
PTP: protein tyrosine phosphatase
*PTPN1*: protein tyrosine phosphatase non-receptor type 1
pTyr: phosphotyrosine
ROS: reactive oxygen species
TNFα: tumor necrosis factor α
WT: wildtype
